# Editorial Comment to Prostate carcinoma with neuroendocrine differentiation successfully treated by early detection with imaging examination

**DOI:** 10.1002/iju5.12235

**Published:** 2020-11-03

**Authors:** Teruo Inamoto, Haruhito Azuma

**Affiliations:** ^1^ Department of Urology Osaka Medical College Takatsuki Osaka Japan

Prostate carcinoma with neuroendocrine differentiation (NECAP) is thought to be a highly malignant subtype of castrate‐ resistant prostate cancer (CRPC) associated with resistance to androgen deprivation therapy (ADT), where cancer cell results in the transdifferentiation into NECAP phenotype as a sequel to prolonged ADT use. Usually, prostate‐specific antigen (PSA) excretion from the NECAP tissue is scarce, making initial detection by PSA screening test complicated. In this case, Kobayashi *et al*. could diagnose potential prostate cancer with extremely low PSA value using conventional magnetic resonance imaging.^1^ Apart from the usual imaging study, computed tomography with Ga‐[DOTA‐Tyr]‐octreotate (Ga‐ DOTA‐TATE), a somatostatin analog that binds somatostatin receptor 2 with high affinity plays a role in evaluating the presence and/or extent of NECAP.^2^ Hence, NECAP that presents in the heterogeneous tissue of the prostate gland should be differentiated from the usual adenocarcinoma. The complete genomic landscape of NECAP along with the impact on downstream transcriptional profile remains to be elucidated.^3^ Most recently, Aggarwal *et al*. exhibited that NECAP was significantly less likely to have amplification of androgen receptor (AR) or an intergenic AR enhancer locus, and demonstrated lower expression of AR and its downstream transcriptional targets using whole‐genome sequencing method.^3^

## Conflict of interest

The authors declare no conflict of interest.
